# Medroxyprogesterone Acetate Alters Mycobacterium Bovis BCG-Induced Cytokine Production in Peripheral Blood Mononuclear Cells of Contraceptive Users

**DOI:** 10.1371/journal.pone.0024639

**Published:** 2011-09-08

**Authors:** Léanie Kleynhans, Nelita Du Plessis, Gillian F. Black, André G. Loxton, Martin Kidd, Paul D. van Helden, Gerhard Walzl, Katharina Ronacher

**Affiliations:** 1 Division of Molecular Biology and Human Genetics, MRC Centre for Molecular and Cellular Biology, DST/NRF Centre of Excellence for Biomedical TB Research, Faculty of Health Sciences, Stellenbosch University, Tygerberg, South Africa; 2 Department of Statistics and Actuarial Sciences, Stellenbosch University, Stellenbosch, South Africa; Universidade Federal de Minas Gerais, Brazil

## Abstract

Most individuals latently infected with *Mycobacterium tuberculosis* (*M.tb*) contain the infection by a balance of effector and regulatory immune responses. This balance can be influenced by steroid hormones such as glucocorticoids. The widely used contraceptive medroxyprogesterone acetate (MPA) possesses glucocorticoid activity. We investigated the effect of this hormone on immune responses to BCG in household contacts of active TB patients. Multiplex bead array analysis revealed that MPA demonstrated both glucocorticoid and progestogenic properties at saturating and pharmacological concentrations in peripheral blood mononuclear cells (PBMCs) and suppressed antigen specific cytokine production. Furthermore we showed that PBMCs from women using MPA produced significantly lower levels of IL-1α, IL-12p40, IL-10, IL-13 and G-CSF in response to BCG which corresponded with lower numbers of circulating monocytes observed in these women. Our research study is the first to show that MPA impacts on infections outside the genital tract due to a systemic effect on immune function. Therefore MPA use could alter susceptibility to TB, TB disease severity as well as change the efficacy of new BCG-based vaccines, especially prime-boost vaccine strategies which may be administered to adult or adolescent women in the future.

## Introduction

Tuberculosis (TB) caused by *Mycobacterium tuberculosis (M.tb)* is a major health problem and the World Health Organization has estimated a global incidence of 9.4 million TB cases and 1.6 million TB related deaths every year [1]. South Africa and Swaziland are the two countries which have the highest incidence at 960 and 1200 per 100 000 population per year, respectively. Even though most individuals infected with *M.tb* contain the infection by maintaining a balance of regulatory and effector immune responses, this balance can be influenced by steroid hormones such as glucocorticoids (GCs) [2]. It has been shown that stress-induced activation of the hypothalamus-pituitary-adrenal axis causes reactivation of TB in mice [3] and that administration of exogenous GCs induces reactivation of TB in animal models [4], [5] and increases the risk of developing TB in humans [6]. Furthermore endogenous concentrations of the GC cortisol have been shown to inhibit mycobacterial antigen-induced cell proliferation and IFNγ production in peripheral blood mononuclear cells (PBMCs) [7].

Medroxyprogesterone acetate (MPA) is used as a three month injectable progestin-only contraceptive and is the most commonly used contraceptive in South Africa and other TB endemic areas. MPA is freely available at public health care clinics in South Africa and is favored by women and health care workers because it is administered only four times a year. MPA is pharmacologically unique compared to other synthetic progestins (such as the 2 month injectable contraceptive norethisterone (NET)) as it binds with high affinity not only to the progesterone receptor (PR), but also to the glucocorticoid receptor (GR) [8] and can alter the transcription of GR-regulated genes [9]. Due to its GC activity it is possible that doses of MPA used for endocrine therapy could have significant immune modulatory effects and impact on susceptibility as well as clinical manifestation of infectious diseases. There is evidence that MPA increases susceptibility to vaginal simian-human immunodeficiency virus transmission and suppresses antiviral immune responses in Rhesus Macaques [10]. Similarly a study in mice found that MPA treated animals had increased vaginal infectability with herpes simplex virus (HSV)-2 [11]. In women an association was found between MPA use and viral shedding of HIV and HSV, from vaginal epithelium cells [12], [13]. Another study reported a significant association between MPA use and the acquisition of sexually transmitted bacterial infections [14]. Studies on whether MPA use itself increases the risk of acquiring HIV are conflicting [15]–[17]. Recently, MPA use was associated with a significantly higher risk of acquiring HIV, but was not associated with disease progression of HIV [18], [19].

The effect of hormone based contraceptives on immune responses in the context of mycobacterial infections has never before been investigated. This is surprising as MPA is mainly used in low socioeconomic areas with a high TB burden. Furthermore MPA is the recommended contraceptive for active TB patients as anti-TB drugs like rifampicin upregulate P450 cytochromes which rapidly metabolize estradiol containing contraceptives, rendering them ineffective [20]. This study is the first to show that MPA alters the secretion of several cytokines in response to BCG in vitro and changes the BCG-induced memory immune response in MPA users compared to non-contraceptive users.

## Materials and Methods

### Ethics Statement

The Ethics Committee of the University of Stellenbosch (N05/11/187) and the City of Cape Town City Health approved the protocols for the study, which was conducted according to the Helsinki Declaration and International Conference of Harmonisation guidelines. Written informed consent was obtained from all study participants.

### Study subjects

For this cross-sectional case control study, we randomly recruited female household contacts (HHCs) of active TB patients between the ages of 15 and 45, who were enrolled at a TB Clinic in the Ravensmead/Uitsig area of Cape Town during 2008. Pulmonary TB index cases were self-reporting with a first episode of TB and had two sputum smears positive for acid fast bacilli. HHCs were defined as individuals living in the same house as an adult pulmonary TB patient who was diagnosed not more than 2 months before recruitment of the HHC. HHCs were tuberculin skin test (TST) positive with an induration of ≥10 mm 48–72 hours after an intradermal injection with *M.tb* purified protein derivative. Study participants were not taking any steroid treatment other than contraceptives at the time of recruitment. HIV positive, pregnant and sterilized women, women that used contraceptives other than MPA and those who's PBMCs did not produce IFNγ in response to BCG were excluded from the study. Two hundred and seventy-four women were recruited of which 111 were using contraceptives (40.5%). The majority of contraceptive users (65/111; 59.3%) used MPA; 14/111 (12.4%) used NET; 14/111 (12.4%) used combined oral contraceptives; 14/111 (12.4%) had been sterilized and 4/111 (3.5%) used an intra-uterine device. The mean age of MPA users and non-contraceptive users was 28±7.03 and 26±8.85 years respectively and the mean duration of MPA use 33±36 months. For the cytokine analysis, PBMCs from a subset of participants were used as indicated in the figure legends. The mean age of MPA users in this subset was not significantly different from controls (MPA users: 29±6.4, controls: 27±5.1) and the mean duration of MPA use was 39±39 months. These study participants were from different households and not related to each other with exception of two sisters. However one of them was a MPA user and the other was part of the control group. Therefore the cytokine expression patterns in the two different groups cannot be attributed to consanguinity. At time of recruitment duration of exposure of study participants to the TB patients was recorded. The majority of MPA users as well as the majority of controls spent more than 12 hours per day with the TB index case and there was no significant difference in exposure score between the two groups.

### Isolation and stimulation of PBMCs

PBMCs were isolated from whole blood using Histopaque (Sigma-Aldrich, SA) density gradient centrifugation. Cells were cultured at a density of 1×10^6^ cells/well in 24-well tissue culture plates (Greiner Bio-one, North Carolina, USA). PBMCs were cultured as unstimulated controls and with Pasteur BCG (5×10^5^ CFU/ml, MOI 1∶5) in the presence and absence of cortisol (hydrocortisone), medroxyprogesterone 17-acetate (MPA) and 4-pregnene-3,20-dione progesterone (Sigma-Aldrich). Prior to use in culture the hormones were dissolved in ethanol (Merck, New Jersey, USA) and stored at −20°C. BCG cultures were grown in Difco Middlebrook 7H9 (BD Pharmingen, San Diago, USA), supplemented with 0.2% glycerol (Sigma), 0.05% Tween 80 (Sigma) and 10% Middlebrook oleic acid albumin dextrose catalase (OADC) enrichment (BD). Aliquots of BCG cultures at logarithmic phase were frozen at −80°C in 10% glycerol (Sigma). The number of viable bacteria was assessed by thawing a frozen aliquot and plating serial dilutions on Middlebrook 7H11 (BD) agar plates. Unstimulated controls and test cultures were exposed to equal concentrations of ethanol with the final concentration not exceeding 0.1%. Phytohaemagglutinin (PHA, Sigma-Aldrich) was included as positive control. The plates were incubated at 37°C and 5% CO_2_ for six days. Supernatants were harvested on day three and day six post stimulation and frozen at -80°C until analysis.

### Quantification of cytokine levels by ELISA and Multiplex bead array

The IFNγ levels in the culture supernatants were quantified by ELISA according to a protocol previously described [21], however with different antibodies (mouse anti-human IFNγ monoclonal antibody/biotinylated detection antibody (BD) and detection solution (Streptavidin-peroxidase together with *ο*-phenylenediamine (Sigma-Aldrich)). The standard curve ranged from 31 pg/ml to 4000 pg/ml. Diluted (1∶10) whole blood taken from a healthy volunteer was stimulated with PHA (10 µg/ml) for four days and the undiluted culture supernatant included on all plates as an internal control.

Human 21-plex Luminex assays (Merck-Millipore, Missouri, USA) were used to simultaneously quantify the levels of the following cytokines in the day three culture supernatants: interleukin (IL)-1α, IL-1β, IL-2, IL-4, IL-5, IL-6, IL-8 (CXCL8), IL-10, IL-12p40, IL-13, IL-17, interleukin 1 receptor antagonist (IL-1ra), epidermal growth factor (EGF), granulocyte colony stimulating factor (G-CSF), granulocyte monocyte stimulating factor (GM-CSF), interferon (IFN)γ, interferon inducible protein 10 (IP-10 or CXCL10), monocyte chemotactic protein-1 (MCP-1 or CCL2), macrophage inflammatory protein (MIP)-1β (CCL4), transforming growth factor (TGF)α and tumor necrosis factor (TNF)α. Human 29-plex Luminex assays (Merck-Millpore) were used to measure the levels of IL-1α, IL-1β, IL-2, IL-4, IL-5, IL-6, IL-7, IL-8, IL-10, IL-12p40, IL-12p70, IL-13, IL-15, IL-17, IL-1ra, soluble CD40 ligand (sCD40L), EGF, eotaxin (CCL11), fractalkine, G-CSF, GM-CSF, IFNγ, IP-10, MCP-1, MIP-1α (CCL3), MIP-1β, TGFα, TNFα and vascular endothelial growth factor (VEGF) in the day six culture supernatants. The assay was done according to manufacturer's instructions and samples were evaluated in duplicate.

The cytokine concentrations in the unstimulated and BCG stimulated (±cortisol, MPA and progesterone) PBMC culture supernatants were measured on a Bio Plex platform (Bio Plex^TM^, Bio Rad Laboratories). Two quality controls included in each kit were run in duplicate on each plate. Levels of all analytes in the quality controls were within the expected ranges. A standard curve ranging from 3.2 pg/ml to 10 000 pg/ml was used for all cytokines. Bio-Plex Manager Software, version 4.1.1, was used to analyze the data.

### Data and Statistical analysis

For the multiplex bead array assay, cytokine levels below the lowest and above the highest concentration of the standard curve were extrapolated by the Bio-Plex Manager software and are reported here. Luminex data were analyzed by a mixed model repeated measures one-way analysis of variance (ANOVA) with a Fisher LSD Post-Hoc test. Luminex results are shown as least-squares (LS) means with 95% confidence intervals (CI). A p-value≤0.05 (when compared to the *M.bovis* BCG only responses) was accepted as significant. Data analysis was done using Statistica 9, Statsoft (Ohio, USA). Unbiased clustering of cytokine secretion of MPA users and non-contraceptive users and generation of a heat map was done using Qlucore Omics Explorer (Lund, Sweden).

## Results

### MPA and cortisol inhibit IFNγ production in BCG-stimulated PBMCs in vitro

**Figure 1:**
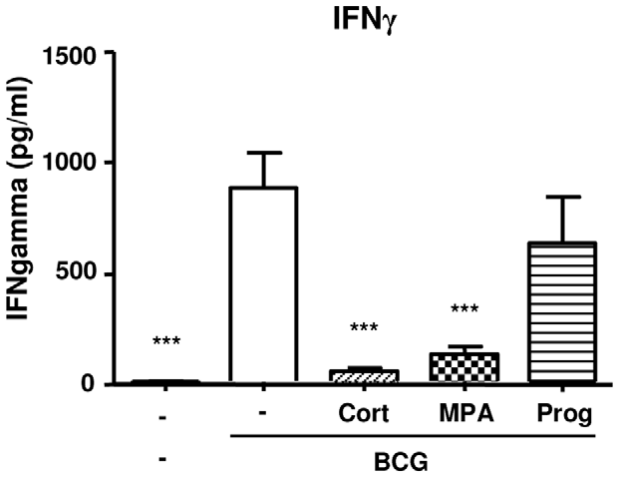
The effect of cortisol (cort), medroxyprogesterone acetate (MPA) and progesterone (prog) on BCG induced IFNγ responses of PBMCs of non-contraceptive users. PBMCs of household contacts of active TB cases were cultured with AIM-V medium only (unstimulated controls), incubated with cort, MPA and prog alone (not shown) or *M.bovis* BCG (5×10^5^ CFU/ml) in the presence and absence of the three hormones (final concentration of 10^−5^ M). IFNγ concentrations were measured by ELISA in the supernatants collected three days post stimulation (n = 29). Differences among stimulations were determined by one-way ANOVA with the Bonferroni Post-Hoc test and the data represented as mean ± SEM. * p<0.05, **p<0.01, ***p<0.001 compared to BCG alone.

Given the importance of IFNγ in protective immunity to TB we investigated whether high concentrations of MPA influence IFNγ production of BCG stimulated PBMCs in vitro. We compared MPA to cortisol and progesterone as these hormones bind to the GR and PR respectively. As mentioned previously MPA can bind and activate both steroid receptors. Furthermore we used saturating ligand concentrations (10 µM) to discount the different binding affinities of the hormones to the respective receptors. Supernatants of PBMCs stimulated with BCG in the presence and absence of the three hormones were collected after three (n = 29) and six days (n = 35) of culture and stored at −80°C until ELISA analysis. Our ELISA results indicated that cortisol nearly abolished BCG-induced IFNγ response in the PBMC supernatants after three (p<0.001; figure 1) and six days (p<0.001; data not shown). MPA inhibited the IFNγ responses to a similar extent as cortisol (p<0.001). In contrast to MPA and cortisol, progesterone did not down regulate the secretion of IFNγ three days post stimulation, however, at six days post stimulation, progesterone significantly reduced IFNγ production, (p<0.01), although not to the same extent as MPA and cortisol. IFNγ concentrations were not detectable in supernatants of PBMCs cultured in the presence of the three hormones alone; while high levels of IFNγ were detected in the supernatants of PBMCs stimulated with the mitogen PHA at both three (2375.48 pg/ml) and six days (2476.73 pg/ml) (data not shown).

### MPA, cortisol and progesterone differentially alter BCG-induced secretion of cytokines in vitro

To determine the effect of cortisol, MPA and progesterone on the secretion of other cytokines and chemokines in addition to IFNγ we used a Multiplex bead array assay. Our Multiplex data revealed that the cytokines, chemokines and growth factors could be divided into four distinct groups; those not changed by any of the three hormones, those influenced by MPA and cortisol, those influenced by MPA, cortisol and progesterone and those influenced by cortisol only (table 1).

**Table 1:** The effect of cortisol, MPA and progesterone on cytokine production three and six days post stimulation of PBMCs with BCG.

	BCG	BCG+Cort	BCG+MPA	BCG+Prog
**Three days post stimulation**				
**IL-6**	8117.2±1146.1	4454.1±.6 ***	5910.6±1160.2 *	7736.1±1266.0
**IL-2**	42.2±11.3	11.6±12.1 *	7.9±11.7 **	24.4±14.8
**IFNγ**	778.6±171.1	32.3±188.4 **	67.8±179.2 **	644.6±49.0
**IL-5**	12.3±3.0	0.2±3.3 **	0.3±3.1 **	6.1±4.4
**GM-CSF**	295.7±67.3	78.5±69.6 *	135.9±68.5 *	182.9±77.5
**MIP-1β**	5253.4±1359.8	3487.8±1380.0 **	3910.1±1370.2 *	4748.8±1447.7
**G-CSF**	597.9±307.5	1285.6±311.8 ***	1146.9±309.7 ***	897.6±326.5
**IL-1ra**	1197.4±357.8	−412.8±377.1 ***	−31.1±367.4 ***	365.1 ± 441.3 *
**IL-1β**	740.8±186.7	304.7±191.8 ***	605.6±189.3	596.9±208.8
**IL-8**	7863.2±1121.2	6772.4±1137.0 *	7328.2±1129.3	8307.5 ± 1190.1
**Six days post stimulation**				
**IL-1α**	676.5±99.2	87.3±103.8 ***	169.1±103.8 ***	432.7±131.7
**IL-1β**	476.4±113.5	98.2 ± 117.3 **	227.3±117.3 *	300.1±138.8
**IL-1ra**	743.3±124.3	−55.7±130.3 ***	66.2±130.3 ***	487.7±166.5
**IL-6**	6744.7±1166.7	3550.7±1179.8 ***	4956.3 ± 1179.8 **	7635.0±1250.7
**TNFα**	2557.3±563.3	316.8±589.9 **	482.6±589.9 **	1192.4±752.1
**IL-5**	17.1±4.1	1.8±4.3 **	1.8 ± 4.3 **	10.1±5.3
**IL-17**	46.0±8.9	5.6±9.3 ***	8.2±9.3 **	31.3 ± 11.8
**IP-10**	2472.6±2052.4	9565.7±2108.9 ***	8672.5±2108.9 **	4776.7±2423.8
**IL-12p40**	205.5 ± 30.9	24.4±32.2 ***	42.5±32.2 ***	114.3±40.1 *
**IFNγ**	2890.1±302.3	95.4±316.6 ***	318.9±316.6 ***	1805.1 ± 403.8 *
**IL-13**	253.1±29.6	4.0 ± 31.1 ***	6.9±31.1 ***	88.0±41.2 **
**sCD40L**	39.6±7.4	−9.9 ± 7.8 ***	−15.1±7.8 ***	−6.7±9.7 ***
**GM-CSF**	455.8±72.9	68.6±75.6 ***	110.7 ± 75.6 ***	217.3±91.2 *
**G-CSF**	799.2±382.9	1547.5±387.4 ***	1371.8±387.4 **	1292.9±411.2 *

Cytokine levels of the unstimulated supernatants were subtracted from the BCG ± hormone stimulated supernatants as measured by multiplex bead array assay. Data were analyzed by mixed model repeated measures ANOVA (LS means ± SEM).

*p<0.05,

**p<0.01,

***p<0.001 compared to BCG alone.

**Figure 2:**
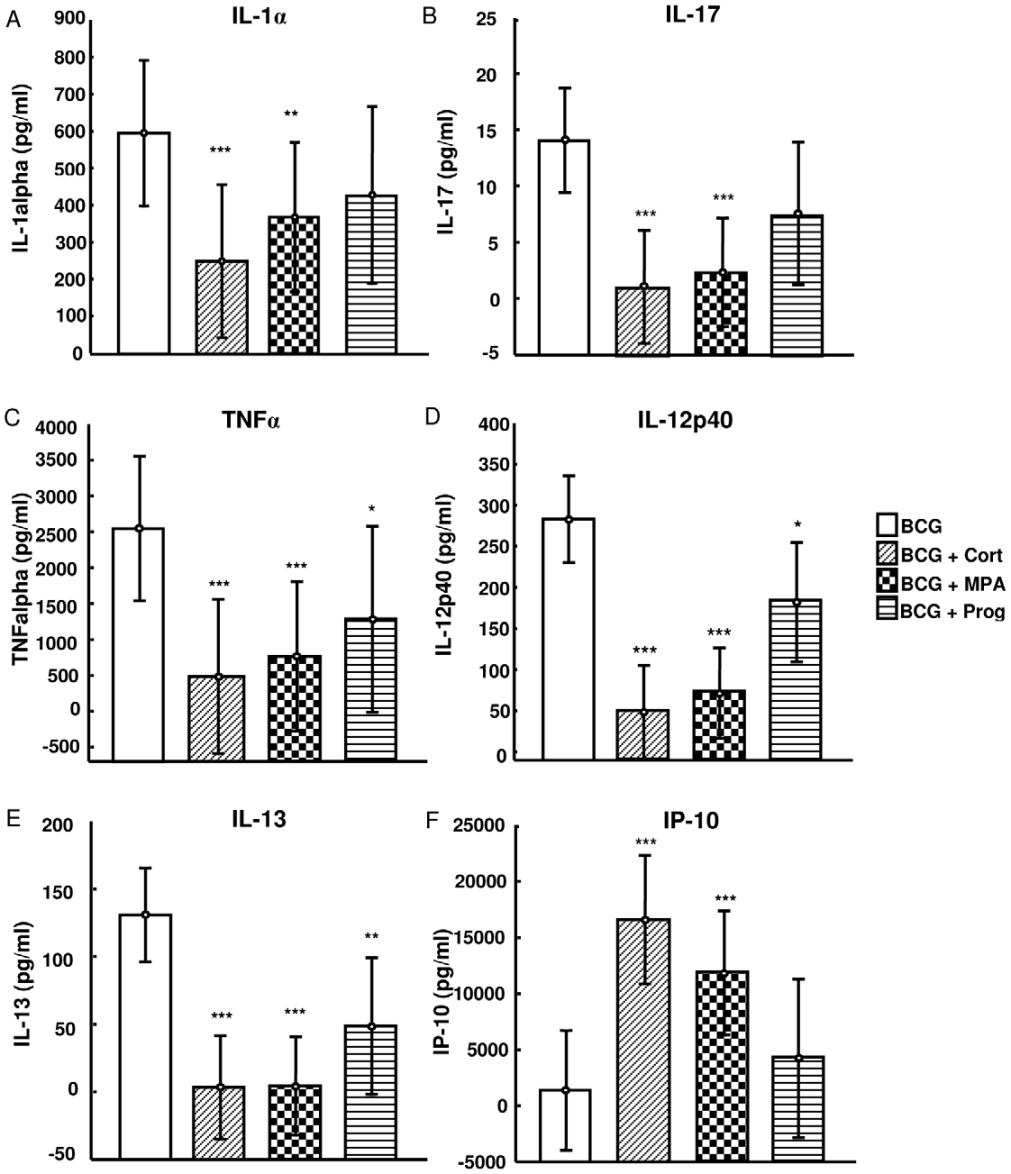
The effect of cortisol, MPA and progesterone on cytokine production three days post stimulation of PBMCs with BCG. Cortisol, MPA but not progesterone altered the expression of IL-1α (A) and IL-17 (B) three days post stimulation of PBMCs from household contacts (non-contraceptive users, n = 11). The BCG-induced expression of TNFα (C), IL-12p40 (D) and IL-13 (E) was altered by cortisol, MPA and to a lesser extent progesterone. Cortisol and MPA also up-regulated the expression of IP-10 (F). These cytokine levels were measured by multiplex bead array assays. Cytokine levels of the unstimulated supernatants were subtracted from the BCG ± hormone stimulated supernatants. Data were analyzed by mixed model repeated measures ANOVA (LS means, 95%CI). * p<0.05, **p<0.01, ***p<0.001 compared to BCG alone.

Three days post stimulation (n = 11) the secretion of IL-10, EGF and MCP-1 was not influenced by any of the hormones (data not shown) whereas the secretion of IL-1α, IL-17 (figure 2 A and B), IL-6, IL-2, IFNγ, IL-5, GM-CSF and MIP-1β (table 1) was significantly down-regulated by cortisol and MPA, but not progesterone. Cortisol and MPA were also able to up-regulate the production of IP-10 (figure 2 F) and G-CSF (table 1). Progesterone inhibited the day three secretion of TNFα, IL-12p40, IL-13 (figure 2 C-E) and IL-1ra (table 1), but to a lesser extent than cortisol and MPA. Secretion of IL-1β and IL-8 was influenced by cortisol only with both cytokines being down-regulated (table 1).

At day six (n = 10), cytokines, chemokines and growth factors could be divided into the same groups described for day three but the distribution of cytokines within those groups was different. The production of EGF, MCP-1, MIP-1β and IL-8 was not influenced by the three hormones (data not shown). Levels of IL-1α, IL-1β, IL-1ra, IL-6, TNFα, IL-5 and IL-17 (table 1) were down-regulated by cortisol and MPA, but not progesterone, while IP-10 was significantly up-regulated by cortisol and MPA (table 1). The secretion of IL-12p40, IFNγ, IL-13, sCD40L, GM-CSF was significantly down-regulated by cortisol, MPA and progesterone; whereas the production of G-CSF was significantly up-regulated by the three hormones (table 1). These results indicate that at high concentrations the synthetic progestin, MPA, mimics cortisol instead of progesterone.

### MPA, cortisol and progesterone differentially affect BCG-induced cytokine production at pharmacological doses

**Figure 3:**
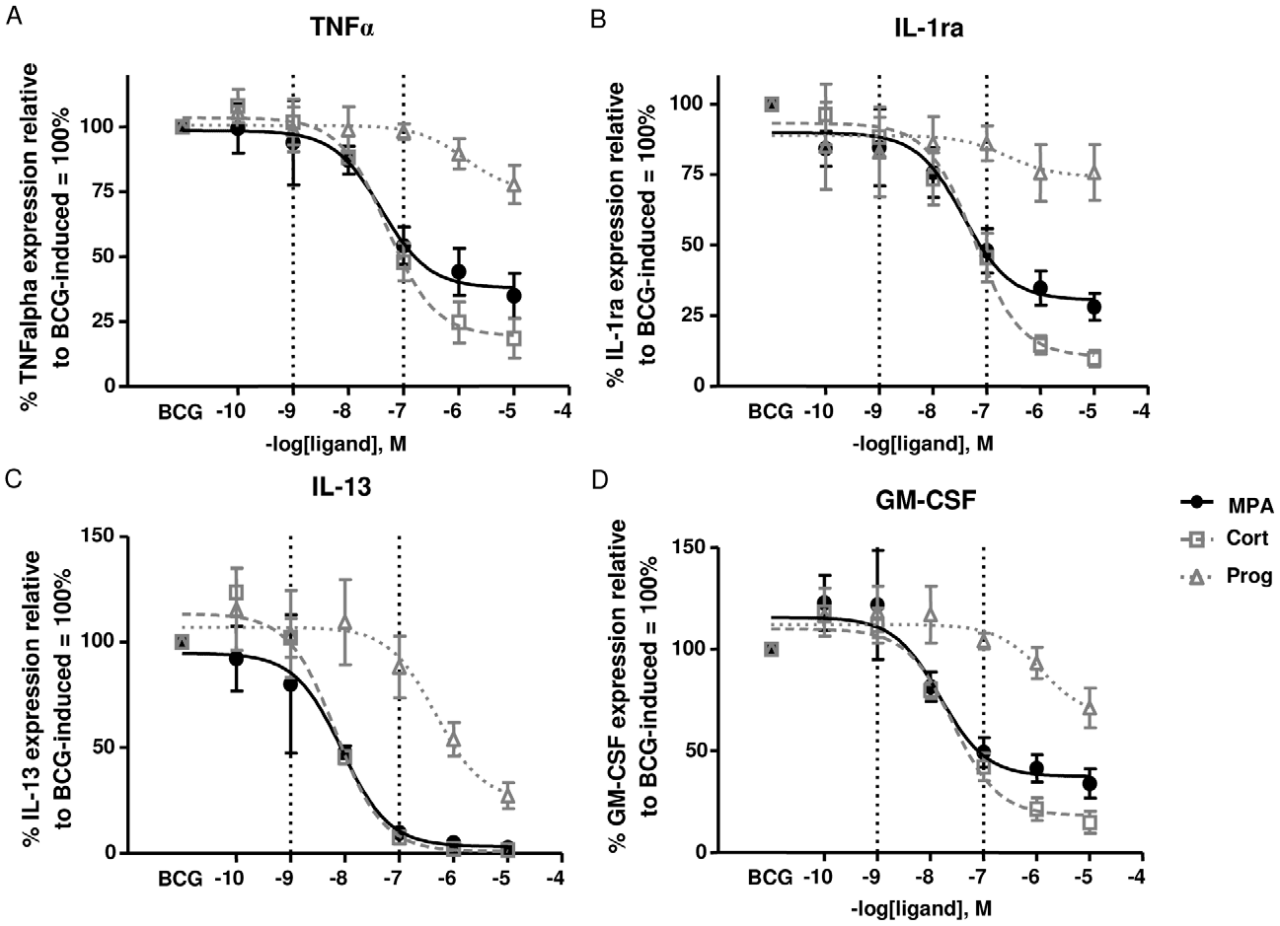
Dose-response curves of cortisol, MPA and progesterone on BCG-induced cytokine expression. Culture supernatants of PBMCs stimulated with a concentration of 5×10^5^ CFU/ml BCG in the absence and presence of the three hormones were collected six days post stimulation (n = 4). TNFα, IL-1ra, IL-13 and GM-CSF (A-D) levels were measured using a Multiplex bead array assay. Hormone stimulated responses are depicted as percentages of the BCG only (100%) responses. Dotted lines indicate the range of MPA serum concentrations measured in contraceptive users. Dose-response curves were generated using GraphPad Prism 5 (nonlinear regression curve) and data are presented as mean ± SEM. Data of four study participants were analyzed to obtain IC_50_ and EC_50_ values.

We found that high concentrations (10 µM) of cortisol, MPA and progesterone alter the secretion of cytokines produced by PBMCs stimulated with BCG. As such high concentrations do not reflect the pharmacological doses of MPA in contraceptive users, dose response experiments were done to determine the effect of the hormones on cytokine production at pharmacological concentrations in vitro. Serum concentrations of MPA in contraceptive users have been shown to range from 0.1 nM to 60 nM [22]–[25]. PBMCs of non-contraceptive users (n = 4) were stimulated with increasing concentrations of the three hormones and the relative potency and efficacy of each steroid to inhibit or enhance the BCG-induced secretion of the cytokines were determined (Table 2). At nanomolar concentrations MPA, cortisol and progesterone did not alter the BCG-induced secretion of IFNγ as determined by ELISA (data not shown), however for TNFα, IL-1ra, IL-13 and GM-CSF MPA mimicked the effect of cortisol (figure 3; pharmacological concentrations of MPA are indicated by the dotted lines). MPA inhibited the production of TNFα (IC_50cort_ = 5.0×10^−8^ M; IC_50MPA_ = 4.0×10^−8^ M), IL-1ra (IC_50cort_ = 5.9×10^−8^ M; IC_50MPA_ = 7×10^−8^ M), IL-13 (IC_50cort_ = 6.9×10^−9^ M; IC_50MPA_ = 8.8×10^−9^ M) and GM-CSF≈(IC_50cort_ = 2.6×10^−8^ M; IC_50MPA_ = 1.5×10^−8^ M) to the same extent as cortisol (Table 2). The effect of MPA and cortisol was different to that of progesterone at similar concentrations (p = 0.0571). Compared to progesterone, MPA had a 33-fold higher potency in inhibiting TNFα; a 6-fold higher potency in inhibiting IL-1ra, a 53-fold higher potency in inhibiting IL-13 and an 87-fold higher potency in inhibiting GM-CSF.

**Table 2:** Potency and efficacy of cortisol, MPA and progesterone as determined by the dose response curves.

	IC_50_ values (potency)			Efficacy (% inhibition)		
	Cortisol	MPA	Progesterone	Cortisol	MPA	Progesterone
IL-1α	2.7E-08	3.5E-08	NA	79.9±30.4	68.1±30.8	20.7±11.6
IL-1β	2.5E-08	9.0E-09	3.3E-08	74.8±31.9	46.9±35.4	7.7±2.6
IL-1ra	5.9E-08#	3.7E-08#	2.4E-07	90.2±5.9	71.9±9.5	24.2±17.1
IL-6	4.2E-08	4.3E-08	1.1E-07	81.2±13.6	67.3±13.9	0
IL-8	7.1E-09	1.2E-05	3.2E-10	2.0±7.5	−2. 0±2.8	−0.3±1.9
TNFα	5.0E-08#	4.0E-08#	1.3E-06	81.5±15.2	65.1±17.5	22.2±14.8
IFNγ	2.6E-07	2.5E-07	8.7E-01	92.2±3.3	46.9±.5	22.4±17.0
IL-12p40	4.7E-07	3.0E-07	4.6E-06	100.0±0.0	50.2±22.2	43.6±20.3
IL-5	9.3E-09	1.0E-08	8.5E-07	78.9±26.6	76.0±31.1	53.5±13.5
IL-13	6.9E-09#	8.8E-09#	4.7E-07	98.4±1.3	97.4±1.9	72.7±12.3
IL-17	3.9E-08	5.9E-08	1.9E-06	88.8±6.2	68.1±6.1	44.5±9.9
GM-CSF	2.6E-08#	1.5E-08#	1.3E-06	89.6±7.9	66.1±14.2	28.9±19.7
EGF	4.4E-09	1.8E-09	3.4E-07	29.6±20.2	5.4±2.3	−5.9±4.9
TGFα	1.2E-08	3.0E-08	4.5E-08	41.4±18.8	39.2±12.3	39.7±4.5
	**EC_50_ values (potency)**			**Efficacy (% induction)**		
G-CSF	2.8E-08	3.5E-08	0	67.0±8.6	59.1±0.1	49.3±9.5
IP-10	3.4E-08	1.9E-08	9.6E-06*	82.3±17.6	83.5±15.5	87.7±0.3

IC_50_ - concentration at which 50% of cytokine response is inhibited; EC_50_ - concentration at which 50% of cytokine response is induced; # - MPA inhibited the expression of TNFα, IL-1ra, IL-13 and GM-CSF with the same potency as cortisol, which was different to progesterone (p value = 0.0571); * - Progesterone, but not MPA or cortisol, induced the expression of IP-10 (p value = 0.0571). NA - no activity for ligands was determined. Dose-response curves were generated using GraphPad Prism 5 (nonlinear regression curve; n = 4). The efficacy was calculated by subtracting the percentage of cytokine expression of the highest concentration of hormone from the BCG only stimulated (100%, no hormone present) response.

### PBMCs of MPA users and non-contraceptive users produce similar levels of IFNγ in response to BCG

Saturating as well as pharmacological concentrations of MPA altered BCG-induced cytokine production of PBMCs of non-contraceptive users. Our data indicate that MPA mimics the effect of cortisol rather than its intended analog, progesterone, which could have major implications on immune function in MPA users. Therefore we investigated whether the immune response to BCG differs between MPA users and women not using contraceptives. After culturing PBMCs of MPA users and controls with BCG (in the absence of hormone) for three (controls n = 29, MPA n = 8) and six (controls n = 35, MPA n = 15) days we found that MPA users did not produce lower levels of IFNγ at any of the two time points (data not shown), which is consistent with our in vitro findings where MPA only inhibited IFNγ production at high but not at concentrations equivalent to serum levels in MPA users.

### PBMCs of MPA users produce lower levels of IL-1α, IL-12p40, IL-10, IL-13 and G-CSF compared to PBMCs of non-contraceptive users

**Figure 4:**
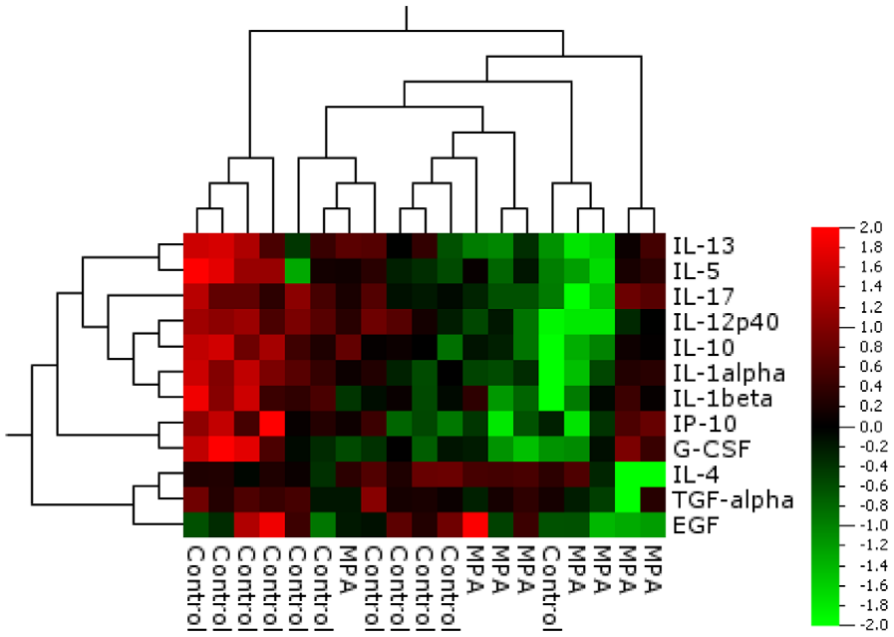
Differential expression of cytokines in supernatants of BCG stimulated PBMCs of MPA users and control participants three days post stimulation. In an unbiased analysis approach using the Qlucore Omics explorer software to generate a heat map, clustering of the participants based on cytokine expression levels coincided with the two study groups (Controls n = 11 and MPA users n = 8). Over-expressed cytokines are depicted in red and under-expressed cytokines in green.

**Figure 5:**
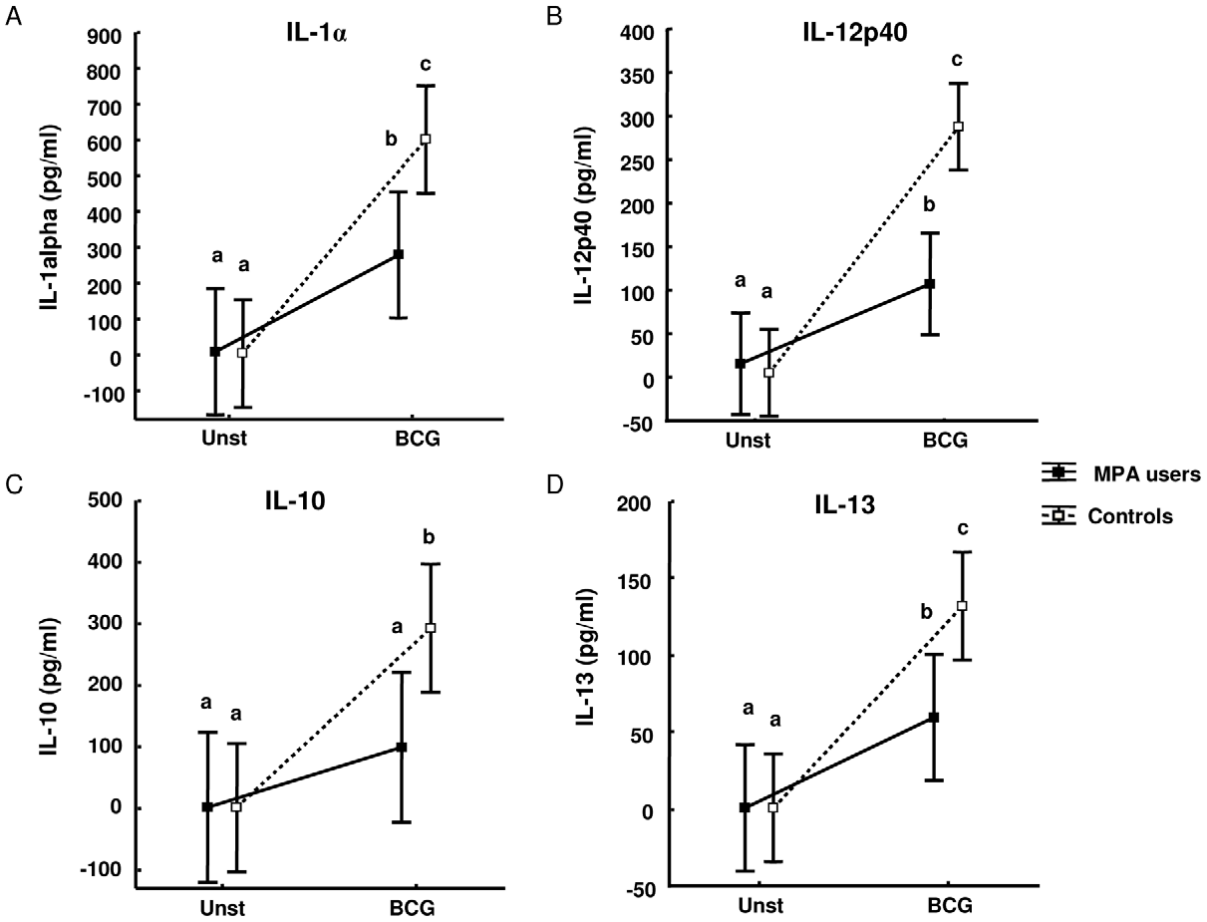
Differential expression of IL-1α, IL-12p40, IL-10 and IL-13 in supernatants of BCG stimulated PBMCs of MPA users and control participants. PBMCs of MPA users (n = 8) and controls (n = 11) were cultured as unstimulated controls and with *M.bovis* BCG. Culture supernatants were collected after three days and cytokine responses were measured by Multiplex bead array assay. Data were analyzed by mixed model repeated measures ANOVA with a Fisher LSD Post-Hoc test and are represented as LS means and 95%CI. The letter a, b and c indicate statistical significance. Values with the same letters are not statistically significantly different from each other.

Since the BCG-induced secretion of other cytokines such as TNFα, IL-1ra, IL-13 and GM-CSF was affected by MPA in vitro at pharmacological concentrations; we investigated whether the levels of these and other cytokines differ in PBMCs of MPA users. Qlucore Omics analysis software was used to generate a heat map of the 21-plex cytokine data from the supernatants of PBMCs of controls (n = 11) and MPA (n = 8) users stimulated with BCG for three days (figure 4). This unbiased analysis approach revealed that MPA users clearly clustered separately from control participants (with exception of one outlier in each group) indicating that the production of various cytokines is affected by MPA use. Neither the outlier in the control nor the outlier in the MPA group showed any obvious differences in demographics, clinical aspects or medicine use compared to the other study participants within the same group. Statistical analysis of the data revealed that PBMCs of MPA users produce significantly lower levels of IL-1α, IL-12p40, IL-10, IL-13 (figure 5 A-D) three days post culture (MPA n = 8 and controls n = 11), as well as lower levels of IL-1α, IL-12p40 and G-CSF six days post culture (MPA n = 10 and controls n = 10; data not shown). There was no significant difference in the age of MPA users and controls, therefore the differences seen in immunological measurements cannot be related to age. Due to the small sample size we were not able to perform accurate correlation analyses of the duration of MPA use with the immunological readouts of the luminex analysis. Since macrophages play a prominent role in either producing these cytokines directly or activating other cells to secrete these cytokines, it raised the question whether the observed effect was due to lower numbers of circulating monocytes in MPA users.

### MPA users have fewer circulating monocytes, but no change in delayed type hypersensitivity responses

**Figure 6:**
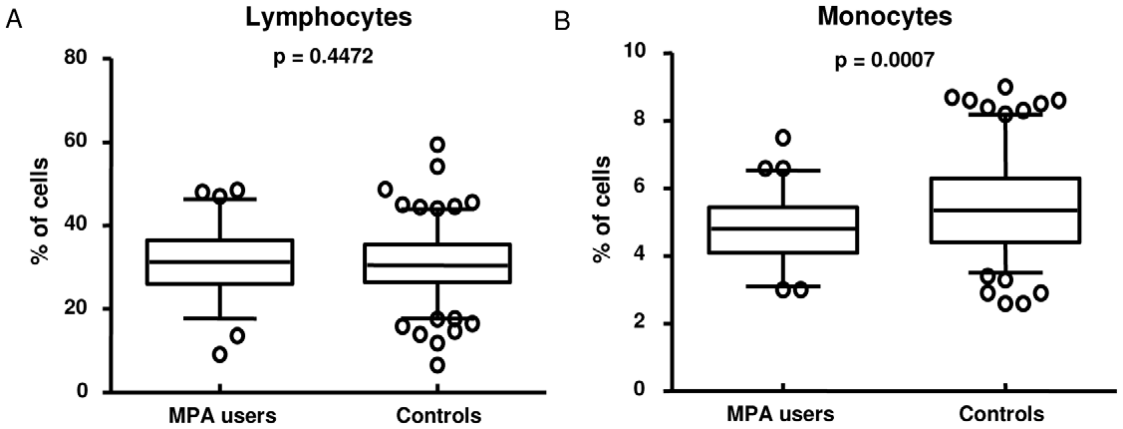
Differential cell counts of MPA users and control participants. Blood was drawn from each study participant (65 MPA users and 163 controls) and the counts determined by the National Health Laboratory Service. Statistical differences were determined by the non-parametric Mann-Whitney test and represented as means and 95%CI.

Full blood counts of 228 HHCs (65 MPA users and 163 Controls) were analyzed and no differences in the white and red cell counts between the two groups of women were observed (data not shown). MPA users had significantly higher levels of haemoglobin and mean corpuscular haemoglobin concentration (data not shown) which is most likely due to amenorrhea frequently experienced by women on MPA [26]. No differences in the numbers of circulating lymphocytes (figure 6 A), basophils, neutrophils and eosinophils were observed (data not shown). Interestingly however, MPA users had significantly lower levels of circulating monocytes (p = 0.0007; figure 6 B), which supports our hypothesis that at least in part a reduced production of IL-1, IL-12p40 and IL-10 can be attributed to fewer macrophages in MPA users. No significant differences were detected in the TST induration of these women when MPA users (19.6 ± 6.4 mm) were compared to controls (21.0±6.8 mm).

## Discussion

In our study population 60% of women using contraceptives choose MPA, which unlike other contraceptives possesses selective glucocorticoid activity and can alter GR regulated genes [9]. Therefore it is extremely important to investigate the potential immune modulatory effects of this synthetic progestin.

This study aimed to determine whether MPA would alter the BCG-specific memory responses of PBMCs of recently exposed HHCs of TB patients. At high concentrations MPA generally mimicked cortisol and unlike progesterone suppressed the production of several cytokines including IL-1α, IL-6 and IL-17. Even at pharmacological concentrations MPA and cortisol but not progesterone, inhibited the production of TNFα, IL-1ra, IL-13 and GM-CSF. Our in vitro findings therefore suggest that in general MPA mimics the effect of cortisol rather than its intended analog progesterone.

GCs affect many aspects of immune cell function and are well known to inhibit the production of Th1 cytokines and cause a shift from a Th1 to a Th2 cytokine response pattern [27]. However the inhibitory effect of GCs goes beyond the traditional pro- and anti-inflammatory division and it has been shown that GCs inhibit the production of IL-2, IFNγ, IL-1β, IL-8, TNFα, IL-4, IL-5, IL-6, IL-10, IL-13, IL-17 as well as G-CSF [28]. Furthermore GCs are able to decrease the activity of the Th1 transcription factor T-box expressed in T cell (T-bet) [29] as well as the Th2 transcription factor GATA-binding protein 3 (GATA-3) [30].

As part of the classical “genomic” GC signaling pathway, GCs mediate their effects by binding to cytosolic GRs which then translocate to the nucleus and bind to the promoter regions of genes regulated by the GR [31]. The anti-inflammatory effects of GCs are mainly mediated by interaction between the ligand activated GR and transcription factors such as NF-κB and AP-1 [32]. Similarly to GCs, MPA has been shown to inhibit the secretion of IL-2 and IL-6 in T cells almost to the same extent as the glucocorticoid dexamethasone, whereas progesterone only slightly inhibited the secretion of these cytokines [33]. During this study we have demonstrated that as with GCs the inhibitory effect of MPA is not restricted to Th1 cytokines alone, but MPA can also impact on Th2 as well as Th17 cytokine responses in vitro.

Ex vivo, we saw that in response to BCG PBMCs from MPA users produced significantly lower levels of IL1α, IL-12p40, IL-10, IL-13 and G-CSF than PBMCs from non-contraceptive users as determined by the Fisher LSD Post-Hoc test. IL-12 plays an important role in the generation of protective immune responses against TB as shown in murine models [34], [35]. T cells rely on IL-12 to differentiate into a Th1 phenotype and produce IFNγ at the site of infection [36]. IL-12 is a heterodimeric cytokine consisting of two subunits, p35 and p40 [37], and is required for optimal differentiation and expansion of activated lymphocytes. It has been suggested that the p40 subunit drives cellular responses [38] as p40 knock-out (KO) mice are more susceptible to *M.tb* than p35 KO mice. IL-1 is one of the most important proinflammatory cytokines and IL-1 type 1 receptor KO mice as well as IL-1α/β KO mice infected with *M.tb*, have significantly larger granulomas with neutrophilic infiltrates in the lungs compared to WT mice [39], [40]. The IL-1 receptor KO mice were highly susceptible to *M.tb* infection and had significantly more bacteria in their lungs, livers and spleens [39]. Susceptibility to *M.tb* was associated with a lack of Th1 responses and impaired granuloma formation. IL-1 is therefore crucial for inflammatory cell recruitment into granulomas and host defense against *M.tb*. Lower levels of BCG-induced IL-12p40 and IL-1α observed in MPA users in our study could therefore suggest that they are more susceptible to *M.tb* and might have a worse outcome of disease.

IL-10 regulates Th1 cell responses by inhibiting the production of IFNγ and IL-12 [41], [42]. Mice deficient in IL-10 have an enhanced ability to control *M.tb* infection as they have significantly lower numbers of bacteria in the lungs and spleens [43]. The increase in protection observed in IL-10 deficient mice was associated with enhanced Th1 responses at the site of disease; however these mice succumb to the disease due to elevated IL-12 and IFNγ levels which result in severe tissue pathology [44]. The increased mortality seen in mice during the absence of IL-10 is not a result of the uncontrolled growth of the bacteria but rather that of unregulated immune responses to the infection. A reduction in IL-10 production will result in a decreased number of circulating Th2 cells leading to less IL-13 being produced; therefore we hypothesize that a reduction of IL-13 could be a result of an indirect effect of the hormone. Lower levels of IL-10 and IL-13, could result in more sever immunopathology in individuals suffering from active TB and using MPA. Taken together it is therefore likely that MPA use will not only have an effect on susceptibility to TB but possibly also TB disease severity.

GC therapy not only inhibits the production of cytokines, but has also been shown to reduce the circulating monocyte population in patients receiving GC therapy [45]. Thus the reduction in numbers of circulating monocytes in women using MPA shown in this study could be attributed to the glucocorticoid activity of this steroid. We could therefore speculate that at least in part the inhibition of IL-12, IL-1α, IL-10 and IL-13 in MPA users can be attributed to lower numbers of circulating monocytes. The reduction of circulating monocytes together with reduced production of IL-1 and IL-12 in response to mycobacterial antigens could increase the susceptibility of MPA users to TB and increase the risk of progression from latent to active disease. For future studies with larger sample numbers it would be particularly interesting to correlate the immunological measurements of MPA users with duration of MPA use.

Our research study is the first to show that MPA impacts on infections outside the genital tract due to a systemic effect on immune function. These findings warrant further investigations into the effect of MPA on susceptibility to TB, disease severity and treatment outcome. Furthermore MPA use could change the efficacy of new BCG-based vaccines and especially prime-boost vaccine strategies which may be administered to adult or adolescent women in the future.
